# Yeast as a Tool for Deeper Understanding of Human Manganese-Related Diseases

**DOI:** 10.3390/genes10070545

**Published:** 2019-07-17

**Authors:** Louise Thines, Antoine Deschamps, Jiri Stribny, Pierre Morsomme

**Affiliations:** Louvain Institute of Biomolecular Science and Technology, Université catholique de Louvain, B-1348 Louvain-la-Neuve, Belgium

**Keywords:** yeast, manganese, disease, antioxidant, cofactor, TMEM165-CDG, Friedreich ataxia, neurodegeneration, Kufor-Rakeb, prion diseases

## Abstract

The biological importance of manganese lies in its function as a key cofactor for numerous metalloenzymes and as non-enzymatic antioxidant. Due to these two essential roles, it appears evident that disturbed manganese homeostasis may trigger the development of pathologies in humans. In this context, yeast has been extensively used over the last decades to gain insight into how cells regulate intra-organellar manganese concentrations and how human pathologies may be related to disturbed cellular manganese homeostasis. This review first summarizes how manganese homeostasis is controlled in yeast cells and how this knowledge can be extrapolated to human cells. Several manganese-related pathologies whose molecular mechanisms have been studied in yeast are then presented in the light of the function of this cation as a non-enzymatic antioxidant or as a key cofactor of metalloenzymes. In this line, we first describe the Transmembrane protein 165-Congenital Disorder of Glycosylation (TMEM165-CDG) and Friedreich ataxia pathologies. Then, due to the established connection between manganese cations and neurodegeneration, the Kufor–Rakeb syndrome and prion-related diseases are finally presented.

## 1. Yeast as a Model to Study Human Pathologies

Yeast is a unicellular eukaryotic organism that is widely used for industrial applications, including controlled fermentation processes and production of recombinant antibodies, vitamins, or drugs [1]. Apart from its use in such industrial processes, yeast plays a key role in fundamental scientific research. Indeed, yeast cells are among the most popular models to explore molecular aspects of fundamental cellular processes, given the high similarity with the ones encountered in plant or animal cells. Part of the knowledge gained when studying yeast can, therefore, be directly transferred to higher eukaryotic organisms. Additionally, being unicellular with short generation time makes yeast both time and cost-efficient to cultivate. Due to its potential as a relevant model organism for higher eukaryotes, a whole molecular toolbox has been developed over decades, thereby facilitating genetics, cell biology, and biochemistry studies. Furthermore, the yeast *Saccharomyces cerevisiae* genome was released in 1996 by Goffeau et al. [2]. Consequently, libraries of single deletant mutants deleted for each of the non-essential genes or overexpressing any gene have been made available. Besides, data obtained by the community using systematic approaches like transcriptomics, proteomics, interactomics, and metabolomics can be easily accessed, thereby facilitating further studies in this organism [3,4,5]. Striking illustrations of how powerful the use of yeast as a model can be are the discoveries of key regulators of the eukaryotic cell cycle [6], of the molecular basis of eukaryotic transcription [7], of how chromosomes are protected by telomeres [8], of the machinery regulating vesicle trafficking [9], and of how cells recycle their own internal structures [10], all leading to Nobel Prizes. 

The availability of both yeast and human genomes led to the identification of yeast orthologs to human proteins, including proteins related to pathologies. Therefore, yeast is widely used as a simplified cellular model of human diseases. A relevant model when yeast cells possess such functional ortholog of the protein linked to the human pathology is generally obtained by deleting the corresponding yeast gene and evaluating the impact of such deletion. In this deletant strain, the impact of disease-causing mutations can then be assessed by heterologously producing wild-type and mutated forms of the human protein. Alternatively, these disease-causing mutations can be studied by introducing the mutation at the corresponding position directly in the yeast gene. Obtaining such yeast models represents a key tool not only to better understand the molecular causes and mechanisms beyond poorly understood human diseases but also to screen for putative modifiers, including drugs, which would suppress the phenotype that corresponds to the pathology [4,11,12,13]. 

## 2. Manganese Homeostasis: From Yeast to Human

### 2.1. Biological Functions of Manganese

Manganese is an essential transition metal required by organisms across every kingdom of life. In biological systems, manganese plays two main roles: (i) to non-enzymatically remove reactive oxygen species (ROS) and (ii) to constitute a cofactor for numerous metalloenzymes involved in a wide range of cellular processes. 

Manganese, when incorporated in non-protein complexes, offers an alternative route than the enzymatic one for the scavenging of ROS, especially superoxides. The exact nature of such Mn-antioxidant complexes has not been defined yet, but Mn-phosphate, Mn-carbonate, and Mn-lactate have been shown to react efficiently with superoxides in vitro [14,15]. These Mn-antioxidant complexes are generally accepted to function as auxiliary protection when enzymatic antioxidants, like superoxide dismutases (Sods), are insufficient or fail. As an illustration of the power of this alternative ROS detoxification pathway, the bacterium *Deinococcus radiodurans*, known to survive under extreme exposure to radiation, accumulates manganese in the millimolar range to overcome the oxidative stress induced by these radiations [16]. Also, the bacterium *Lactobacillus plantarum* lacks antioxidant enzymes and seems to exclusively rely on these Mn-antioxidant complexes for protection against oxidative stress [17]. Majority of our knowledge on manganese-antioxidant complexes comes from the investigation in both bacterial and yeast systems, but such complexes are likely to be encountered in higher eukaryotes [18,19].

Manganese is additionally used as a cofactor by numerous metalloenzymes that are involved in a wide range of cellular processes, including but not restricted to protein glycosylation, DNA biosynthesis, photosynthesis, and enzymatic detoxification of ROS. Manganese-dependent enzymes generally use manganese in a Lewis acid-base reaction or as a reduction/oxidation center to facilitate catalysis. Since Mn-dependent enzymes are present in various cellular compartments, like the nucleus, mitochondria, chloroplasts, cytosol, or Golgi, tightly-controlled manganese homeostasis within the cell is crucial [19]. 

### 2.2. Manganese Transporters in Yeast

**Figure 1:**
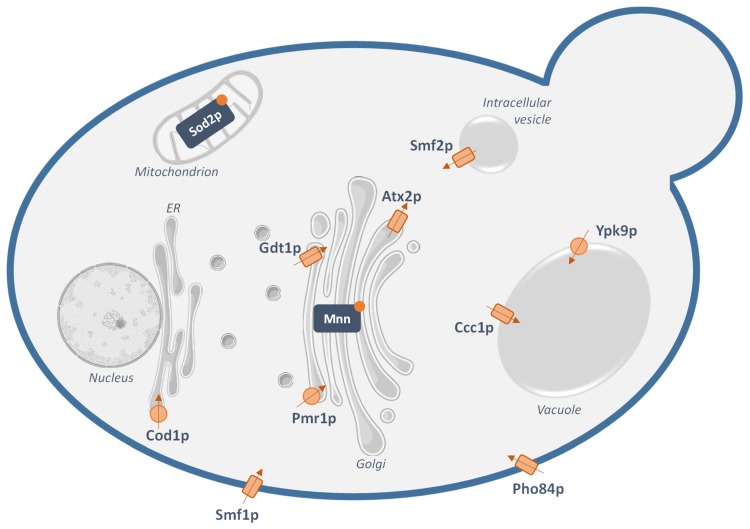
Suggested manganese transport pathways in the yeast *Saccharomyces cerevisiae.* The currently-identified yeast transporters proposed to transport Mn^2+^ are: Smf1p, a plasma membrane Natural Resistance-Associated Macrophage Protein (NRAMP) transporter; Pho84p, a plasma membrane phosphate transporter that can transport Mn^2+^; Pmr1p, a Golgi Ca^2+^ and Mn^2+^-transporting P-type ATPase; Gdt1p, a Golgi Ca^2+^ and Mn^2+^ secondary transporter; Atx2p, a putative Mn^2+^ transporter at the Golgi; Cod1p, a Mn^2+^-transporting P-type ATPase at the endoplasmic reticulum (ER); Smf2p, a NRAMP transporter that localizes in intracellular vesicles; Ypk9p, a P-type ATPase importing manganese in the vacuole; Ccc1p, a vacuolar Mn^2+^ and Fe^2+^ transporter. These transporters ensure proper maintenance of the manganese concentrations in the physiological range and activity of the Mn^2+^-dependent enzymes: the superoxide dismutase Sod2p in the mitochondrial matrix and mannosyltransferases (Mnn) in the Golgi lumen. The arrows represent the putative direction of transport of Mn^2+^, while the rectangles and circles correspond to secondary transporters and ATPases, respectively.

Knowledge about how the yeast *S. cerevisiae* regulates manganese concentrations within the cell has been accumulating over the years (Figure 1) (reviewed in [20,21]). First, manganese influx in yeast occurs via the well-characterized high-affinity Natural Resistance-Associated Macrophage Protein (NRAMP) transporter Smf1p that acts as an Mn^2+^/H^+^ symporter at the plasma membrane [22]. Smf1p is thought not to be the only way of manganese import into the cell. Indeed, the high-affinity cell surface phosphate/proton symporter Pho84p has been shown to contribute to Mn^2+^ uptake in the form of MnHPO_4_ [23]. To now, one cannot exclude that other high-affinity transporters of other metals or other phosphate uptake systems also contribute to cellular manganese intake. 

Once inside the cell, manganese needs to be properly dispatched in the different subcellular compartments where Mn^2+^-dependent enzymes are localized. Another NRAMP transporter, Smf2p, plays a key role in the intracellular distribution of manganese. Unlike Smf1p, Smf2p does not localize to the plasma membrane but to intracellular Golgi-like vesicles. How Smf2p regulates yeast cellular manganese homeostasis from these Golgi-like vesicles is not fully understood yet. One of the proposed models raises the hypothesis that these Smf2p-containing vesicles would act as a transient storage compartment for manganese cations. Hence, once inside the cell, manganese would first converge to these vesicles to be then distributed to the different organelles when needed [24]. How the metal would be delivered from these vesicles to the organelles remains not directly demonstrated. 

Within the secretory pathway, manganese serves as a cofactor for numerous mannosyltransferases involved in protein glycosylation. In this frame, the P-type ATPase Pmr1p is a well-characterized Mn^2+^ transporter at the Golgi membrane that also transports calcium, from the cytosol to the Golgi lumen. The physiological role of Pmr1p is generally described as providing the Golgi-localized Mn^2+^-dependent enzymes with their cofactor as well as detoxifying the cell in case of excess of manganese. Indeed, once in the Golgi lumen, manganese cations can be directed out of the cell via the secretory pathway [25,26]. Recently, Gdt1p has been identified as another transporter of both manganese and calcium presumably from the cytosol to the Golgi lumen, most likely in exchange of protons [27]. Gdt1p and Pmr1p would then have similar physiological roles and would act in concert in a wide range of environmental stresses. Finally, in addition to Pmr1p and Gdt1p, the manganese concentration within the Golgi appears to be controlled by a third transporter: Atx2p. The precise mechanism of transport of Atx2p remains unclear, but indirect data suggest that it would transport Mn^2+^ in the opposite direction than Pmr1p and Gdt1p, i.e., from the Golgi lumen to the cytosol [28]. Still localized in the secretory pathway but in the endoplasmic reticulum (ER) membrane, the P-type ATPase Cod1p, also denominated as Spf1p, would transport both Ca^2+^ and Mn^2+^ from the cytosol to the ER lumen. However, like for Atx2p, the exact substrates and the mechanism of transport still need to be further elucidated [29]. 

Excretion of manganese through Pmr1p and Gdt1p via the secretory pathway is not the only way of detoxification in case of manganese excess. Indeed, the vacuole is a major site for metal-ion sequestration. So far, Ccc1p has been identified as a transporter importing Mn^2+^ in the vacuole for detoxification. While not directly demonstrated, Ccc1p is thought to function as an Mn^2+^-Fe^2+^/H^+^ antiporter [30]. Additionally, the P-type ATPase Ypk9p would also import manganese in the vacuole and, thereby, play a role in the sequestration of divalent metal ions [31]. 

Apart from the Golgi-localized mannosyltransferases, the superoxide dismutase Sod2p that resides in the mitochondrial matrix also requires Mn^2+^ as a cofactor. In yeast mitochondria, manganese is incorporated in low molecular mass metal complexes that may be used to metalate apo-metalloproteins as they fold [32]. However, no manganese importer in the mitochondria has been identified to now. The manganese cations required by Sod2p would then most likely be sent to the mitochondrial matrix by transporters of other divalent metal ions with low affinity for Mn^2+^ or by unidentified Mn^2+^ transporters. Another unknown on how yeast cells regulate manganese homeostasis resides in the fact that no manganese exporter at the plasma membrane has been identified so far. 

### 2.3. From Yeast to Human

Much of our current understanding of manganese homeostasis in humans has been elucidated using the budding yeast *S. cerevisiae*. However, while part of the knowledge accumulated in yeast can be extrapolated to humans, thanks to conserved cellular machinery in these two organisms, notable differences of how manganese concentrations are regulated at the cellular level also occur. Besides, describing manganese homeostasis in human cells represents the additional challenge that the identified manganese transporters are differentially expressed in the different cell types. Among the human transporters involved in manganese homeostasis, some are orthologs to the yeast transporters described above. These human orthologs and their main function are detailed in Table 1.

## 3. Use of Yeast to Elucidate the Molecular Background of Manganese-Related Disorders

### 3.1. Molecular Mechanisms Behind Cellular Manganese Toxicity

Manganese-induced pathologies may result from the presence of mutations in genes coding for one of the human manganese transporters or in any gene coding for a protein that indirectly modulates cellular manganese homeostasis (genetic factor). Alternatively, manganese under- or overexposure that cannot be overcome by these transporters may result in non-physiological manganese distribution within the cell and, hence, trigger the development of disorders (environmental factor). The genetically-encoded mutations or the inadequate Mn^2+^ exposure will consequently affect the cellular manganese homeostasis. Manganese toxicity is mostly associated with an excess of this cation, the deficiency being only rarely encountered in humans. The exact mechanism of manganese toxicity has not been elucidated yet. Nevertheless, previous studies hypothesized that manganese overexposure might lead to mitochondrial dysfunction, thereby impairing energy metabolism. Indeed, manganese is known to specifically accumulate in the mitochondria within the cell. There, manganese inhibits enzymes involved in mitochondrial electron transfer chain [39]. Additionally, Mn^2+^ is known to be mutagenic and has been shown to induce, in yeast, mutations almost specifically within the mitochondrial genome, perhaps by substituting to magnesium at the cofactor binding site of the mitochondrial DNA polymerase or by directly binding to DNA [40,41]. Interestingly, like for Parkinson, manganism, a neurodegenerative disease encountered in populations in contact with manganese overexposure, has been associated with such mitochondrial dysfunction. Besides, and still related to manganism, manganese has been shown to alter the neurotransmitter dopamine release by affecting the trafficking of the dopamine transporter, hence, generating behavioral defects [42]. Mn^2+^-induced neurotoxicity has also been associated with induced misfolding of disease-causing proteins, as is further detailed in the light of α-synuclein and the prion protein. The toxic effects of manganese might also result from the fact that it would impact the homeostasis of other cations, for example, by competing with other substrates for transport, by exerting a regulation action on the transporters of these other substrates, or by modifying their level of expression or trafficking. For example, chronic manganese exposure in rats has been shown to alter the homeostasis of iron [43]. Excess of manganese can also lead to the substitution of other cations acting as a cofactor at the binding site of metalloenzymes, thereby affecting their function. Among others, in rats, Mn^2+^ treatment inhibits the mitochondrial Fe^2+^-dependent aconitase activity, by the replacement of Fe^2+^ by Mn^2+^ [44]. Finally, since manganese cations, when incorporated into complexes, act as non-enzymatic antioxidants, altered homeostasis may lead to affected resistance to oxidative stress. In the light of this manganese toxicity, this section gathers illustrations of how powerful yeast can be to understand manganese-related diseases. However, a full understanding of the development of diseases is generally complex and requires to consider multiple factors. Indeed, while focusing this review on the involvement of manganese in the molecular basis of the pathology, it does not imply that other well-established disease-causing factors are not at stake.

### 3.2. Disorders Related to Altered Bioavailability of Mn^2+^ as Non-Enzymatic Antioxidant

Free radicals are naturally and continuously produced in our cells as a by-product of aerobic metabolism. An excess of these radicals due to altered oxidative stress protection is known to lead to the development of many pathologies, including but not restricted to cancer, Alzheimer disease, or diabetes. Cells protect themselves against oxidative stress via the endogenous activity of the antioxidant superoxide dismutases, glutathione peroxidases, and catalases that act in concert in human cells [45]. Apart from this enzymatic defensive mechanism, it has been shown that manganese, when incorporated in non-enzymatic complexes, can enhance oxidative stress protection in various organisms when enzymatic antioxidants become non-sufficient to counter this stress [18].

While most of the human disorders attributed to altered oxidative stress protection are thought to be caused by a decreased activity of the enzymatic antioxidants, including the mitochondrial manganese-dependent SOD2 [46], one cannot exclude that altered non-enzymatic Mn-antioxidant complexes may at least partly contribute to few pathologies. Besides, in the precise case of disease-related deficiency of the Mn-dependent SOD2, one could consider dietary manganese supplementation at a sub-toxic concentration as a treatment to provide an alternative route to the enzymatic one to cope with oxidative stress. Another aspect in which the non-protein Mn-antioxidants are biologically relevant lies in their role in preventing pathogen invasion. Indeed, after infection, host cells defend themselves by triggering an oxidative burst to challenge the pathogen, leading to an increased level of free radicals in its direct neighborhood, in macrophages, and neutrophils. In complement to this burst, host cells, via the action of the transporter NRAMP1, starve the pathogens of essential metals, including manganese, that would help to deal with this oxidative stress through its antioxidant action [18,47]. In that frame, understanding how these non-enzymatic Mn-antioxidants are formed and to what extent they work is of particular interest.

In yeast, the relevance of manganese as a non-enzymatic antioxidant has been demonstrated by the fact that the growth defect caused by deleting the *SOD* genes in aerobic conditions could be suppressed by supplementing the extracellular medium with manganese [48]. Additionally, extra deletion of the genes coding for the manganese transporters Ccc1p and Pmr1p, both involved in manganese detoxification, has been identified as restoring aerobic growth in a strain deleted for the *SOD* genes, again illustrating the importance of manganese as a backup to the enzymatic protection [26,49]. Interestingly, the amount of manganese required to compensate for the loss of the *SOD* genes is below its toxic threshold, thereby making yeast cells able to benefit from its antioxidant properties without its detrimental side effects. How the non-enzymatic Mn-antioxidants are formed has also been studied in yeast. In that organism, it has been shown that such complexes are not passively and constitutively produced but are tightly regulated. Indeed, their formation is controlled by environmental factors, more precisely through nutrient sensing and subsequent nutrient signaling pathways [50]. The exact nature of these complexes has also been further questioned via spectroscopic approaches. Such analyses led to the detection of manganese associated with inorganic phosphate (Pi) in yeast cells. Interestingly, a correlation could be established between the viability of cells deleted for the *SOD* genes under aerobic conditions and the concentration of the [Mn-Pi] complexes, thereby strengthening the hypothesis that these complexes are indeed involved in protecting cells against oxidative damage [51].

Although Mn-antioxidants have been widely characterized in bacteria and yeast, their contribution to oxidative stress resistance in higher organisms is not clearly understood yet. It is, however, likely that multicellular organisms, including humans, have evolved with analogous or even more complex systems to exploit manganese in order to cope with the production of reactive side-products of the aerobic metabolism [18]. Therefore, further exploration of the identification and function of these complexes, including through the use of yeast, is a key step for a complete understanding of how cells deal with oxidative stress and associated pathologies.

### 3.3. Disorders Related to Altered Bioavailability of Mn^2+^ as a Cofactor

Apart from its role as a non-enzymatic antioxidant, the biological importance of manganese lies in its ability to interact as a cofactor with many proteins to ensure their proper function. As a consequence, unbalanced manganese homeostasis may disturb its interactions with metalloenzymes involved in essential and various cellular processes that would thereby be affected. This latter statement is illustrated in this Section by two disorders, studied through yeast as a model, for which the molecular causes have been assigned to unbalanced bioavailability of Mn^2+^-cofactor: Transmembrane protein 165-Congenital Disorder of Glycosylation (TMEM165-CDG) for the glycosyltransferases involved in protein maturation at the Golgi, and Friedreich ataxia for the superoxide dismutase 2 SOD2 involved in oxidative stress protection in the mitochondrial matrix.

#### 3.3.1. TMEM165-CDG and Mn^2+^-Dependent Glycosyltransferases

Congenital disorders of glycosylation encompass a rapidly growing group of human rare diseases characterized by glycosylation defects [52]. Specific mutations within the gene coding for the human protein TMEM165 have been described, in 2012, as causing a new sub-type of CDG associated with broad and severe symptoms like dwarfism, mental and psychomotor retardation, muscular weakness, and fat excess, among others [53]. TMEM165 belongs to the Uncharacterized Protein Family 0016 (UPF0016), just like its yeast ortholog denominated Gdt1p [54]. Both proteins have been shown to localize at the Golgi membrane [37]. When mutations within TMEM165 are associated with the development of glycosylation defects, the function of any member of the UPF0016 family is unknown. Investigation of the function of these proteins has been first undergone through in silico analyses, from which it appears that the predicted topology of the UPF0016 members is highly similar to that of well-characterized secondary transporters [53,54]. Then, in order to shed light on the functional properties of TMEM165 and its orthologs, the impact of deleting in *S. cerevisiae* the gene coding for its UPF0016 member Gdt1p has been evaluated. In this context, it has been observed that deletion of *GDT1* impairs resistance towards high external concentrations of both Ca^2+^ and Mn^2+^ [27,37]. Interestingly, re-expression of *TMEM165* in the yeast strain lacking *GDT1* could suppress Ca^2+^-related growth defects, thereby showing that Gdt1p and TMEM165 are functional orthologs and validating the use of yeast as a model for this disease [37]. Further studies showed that deletion of *GDT1* modulates the calcium response observed after applying salt stress, leads to an increased cellular Mn^2+^ content, and modulates the activity of the Mn^2+^-dependent enzyme Sod2p [27,55]. These data all strengthen the involvement of the TMEM165 yeast ortholog in both calcium and manganese transport at the Golgi level. The Ca^2+^ and Mn^2+^ transport activity of Gdt1p is further confirmed by its heterologous production in the bacterium *Lactococcus lactis* and by the use of the fluorescent probe Fura-2 that responds to these two cations [27,55]. However, the question of the direction/reversibility of transport of these substrates in yeast remains fully open.

**Figure 2:**
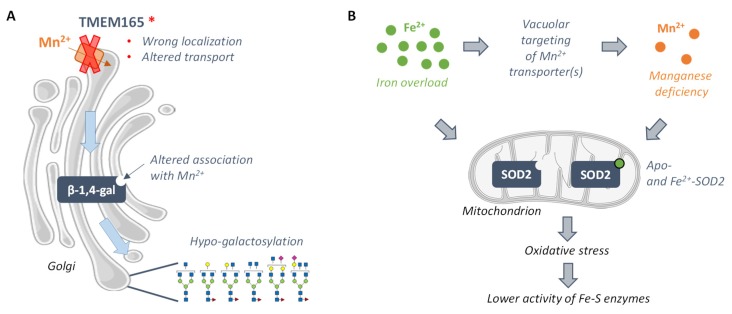
Manganese-related molecular mechanisms beyond the development of Transmembrane protein 165-Congenital Disorder of Glycosylation (TMEM165-CDG) (**A**) and of Friedreich ataxia (**B**). (**A**) Disease-causing mutations within the human gene coding for the Golgi-localized protein TMEM165 are thought to cause its mislocalization or to affect its transport capacity, both leading to disturbed manganese homeostasis in the Golgi lumen. In this compartment, the altered bioavailability of manganese for the Mn^2+^-dependent β-1,4-galactosyltransferase (β-1,4-gal) would affect its enzymatic function and lead to the production of hypo-galactosylated N-glycans. (**B**) Friedreich ataxia is characterized by a decreased production of the mitochondrial frataxin and by iron overload. This metal overaccumulation is, in turn, thought to induce, in yeast, vacuolar targeting of the protein Smf2p, that plays a key role in manganese dispatching within the cell, which, in turn, leads to cellular manganese deficiency. Both manganese deficiency and iron overload drive the formation of the inactive apo- and Fe-bound superoxide dismutase 2 (SOD2), thereby altering oxidative stress protection and leading to decreased activities of the Fe-S enzymes.

Interestingly, similarly to what is detected in patients suffering from TMEM165-CDG, glycosylation defects are observed in the *gdt1Δ* strain when grown in the presence of high external calcium concentration. Both in TMEM165-depleted human cells and in yeast *gdt1Δ* cells grown in the presence of calcium, these glycosylation defects could be suppressed by the addition of manganese in the external medium [55,56,57]. In human and yeast cells, the structure of the defective glycans has been further analyzed by mass spectrometry. In yeast, strong defects in α-1,3- and α-1,2-mannosylation are observed while in human cells, the results highlight strong galactosylation defects, moderate N-acetylglucosaminylation deficiencies, and slight sialylation defects. These defects can be assigned to the malfunction of Golgi Mn^2+^-dependent enzymes: the mannosyltransferases Mnn2p, Mnn5p, Mnn6p, and/or Mnn1p in yeast, and mainly the β-1,4-galactosyltransferase I for the strong galactosylation defects in humans [56,57]. The identification of the enzymes corresponding to the glycosylation defects as Mn^2+^-dependent and the fact that manganese restores glycosylation, both in yeast and human cells, suggest that the molecular causes of TMEM165-CDG could be disturbed manganese homeostasis at the Golgi. Hence, specific mutations within TMEM165 possibly affect the function of the Mn^2+^-dependent glycosyltransferases presumably due to altered association with their cofactor, thereby impairing protein glycosylation and causing the severe symptoms observed among patients (Figure 2A). Of course, as TMEM165 and Gdt1p both seem to transport other cations than manganese, mainly calcium, one cannot rule out the possibility that the glycosylation defects are also partly caused by a disturbed calcium balance at the Golgi. In the light of the molecular causes of the pathology, dietary supplementation of manganese appears as one possibility of treatment for TMEM165-CDG patients in order to restore the activity of these Mn^2+^-dependent enzymes. However, due to the established toxicity of manganese excess, oral galactose intake is considered instead and is shown to significantly improve both biochemical and clinical parameters of the patients, including a decrease in hypo-galactosylated N-glycan structures [58].

Apart from leading to a better understanding of the molecular causes of TMEM165-CDG, the development of the yeast model for the pathology at the molecular level, namely the yeast *gdt1Δ* strain producing the human TMEM165, opens new perspectives for further biochemical characterization of the protein and for exploration of the impact of CDG-causing mutations on the transport properties of TMEM165 and on the resulting glycosylation profile. Due to the need for further optimization of the heterologous production of functional TMEM165 in yeast, such strategy has not been carried out yet, but the corresponding disease-causing mutations have been directly introduced in the gene coding for Gdt1p. The data obtained in yeast suggest that only one of the identified disease-causing mutations leads to activity deficiency. Instead, the pathogenicity of the other identified mutations would result from wrong protein localization, as investigated in human cells [59]. From a more fundamental point of view, mutations in conserved regions of the UPF0016 members are introduced in the yeast Gdt1p. On this basis, it is found that the negatively charged and hydroxyl-containing residues comprised in the two copies of the featured motif of the UPF0016 family are essential for the transport ability of the protein [60].

#### 3.3.2. Friedreich Ataxia and the Mn^2+^-Dependent Superoxide Dismutase 2 SOD2

Friedreich ataxia is a human neurodegenerative and myocardial disease associated with a decreased production of the mitochondrial protein frataxin [61]. Affected patients show symptoms like gait and limb ataxia, hypertrophic cardiomyopathy, and diabetes mellitus [62]. At the cellular level, the function of frataxin is not exactly defined yet. There is, however, evidence that it is involved in iron metabolism, and that it would play a role in iron-sulfur clusters formation and heme biosynthesis [63,64,65,66], as well as in iron storage, among others [67]. Apart from the decreased level of frataxin, the pathology also causes iron overaccumulation and alters the activity of the iron-sulfur-containing enzymes aconitase and succinate dehydrogenase, as observed both in patients [68] and in the mouse model of the disease [69]. Over the last decades, Friedreich ataxia has been extensively studied through the yeast *S. cerevisiae* model that consists of the strain deleted for the gene *YFH1*, coding for the yeast ortholog of frataxin. In this context, it has been observed, like in patients and in the mouse model, that the yeast *yfh1Δ* strain overaccumulates iron and has a decreased activity of iron-sulfur-containing enzymes [70,71]. Besides, it has been verified that human frataxin complements the *yfh1Δ* mutant strain [72].

The molecular defects related to the pathology, namely the decreased activity of the iron-sulfur-containing enzymes, are generally associated with the putative function of frataxin in the biosynthesis of the iron-sulfur cluster. However, in 2006, proteomic analysis of the *yfh1Δ* mutant strain opened new horizons for the molecular understanding of the pathology. This analysis revealed that deletion of *YFH1* causes an increased abundance of several proteins involved in antioxidant defenses, including the manganese-dependent Sod2p, presumably reflecting a situation of oxidative stress in these cells. This oxidative stress is most likely a consequence of the high intracellular level of iron, known as a prooxidant agent. Surprisingly, despite its higher production, the activity of Sod2p was lower than in the wild-type strain. This decreased Sod2p activity is associated with a lower cellular Mn^2+^ content in the *yfh1Δ* background. Additionally, when growing the *yfh1Δ* cells in the presence of manganese, the activity of Sod2p, as well as the ones of the iron-sulfur-containing enzymes glutamate synthase, succinate dehydrogenase, and isopropylmalate dehydratase, was restored. These data indicate that the altered activity of Sod2p is caused by a decreased bioavailability of manganese as a cofactor. In addition to the manganese depletion, iron, when present in excess, may also substitute to manganese at the Sod2p cofactor binding site, also leading to enzymatic inactivity. In turn, the oxidative stress induced by the decreased activity of Sod2p would impair the activity of the iron-sulfur-containing enzymes (Figure 2B) [73,74]. Since such alteration of the activity of the Mn^2+^-SOD2 has also been described in other models of Friedreich disease, this raises the question of dietary manganese or other antioxidant molecules supplementation at a sub-toxic dose as a treatment for the patients [75,76].

The decreased manganese content in the *yfh1Δ* strain, suggested as leading to altered activities of Sod2p and, hence, of the Fe-S-containing enzymes, is hypothesized to be an indirect consequence of iron overload. Indeed, when preventing cellular iron overaccumulation in the *yfh1Δ* background using chelating agents, the activities of both the Mn^2+^-Sod2p and the iron-sulfur-containing enzymes were restored. Additionally, both the manganese content and the activity of Sod2p are also shown to be reduced in the *grx5Δ* yeast strain that is known to overaccumulate iron, thereby confirming that iron overload indeed leads to manganese deficiency [73]. Of course, the next step is to understand how iron overaccumulation alters the cellular manganese content. One hypothesis is that the yeast Smf2p, that localizes to Golgi-like vesicles and is presumably involved in manganese dispatching within the cell, is known to be post-translationally regulated by iron and manganese levels. More precisely, upon metal-replete conditions, Smf2p has been shown to be targeted to the vacuole for degradation. Thus, manganese deficiency in *yfh1Δ* cells may be the consequence of the vacuolar targeting of Smf2p that is required for proper manganese dispatching [77].

Another study on Friedreich ataxia carried out in yeast took advantage of the use of conditional *YFH1* mutants to distinguish the primary defects directly linked to the absence of the protein from the secondary ones induced by the oxidative stress caused by iron deregulation. This study indicates that deregulation of iron metabolism is the primary effect of *YFH1* deletion whereas the increased oxidative damage, the decreased manganese levels, and the altered Sod2p activity are indeed secondary effects and the consequence of iron overload [77]. Besides, while examined here in terms of oxidative stress and altered Mn^2+^ bioavailability, other factors most likely jointly contribute to the development of the pathology.

On a broader scale, studies have reported a decline in the activity of SOD2 during pathologies like cancer, asthma, aging, and transplant rejection [78]. While this decreased enzymatic activity may result from an altered level of expression or post-translational modifications, it can also be the consequence of disturbed manganese homeostasis at the mitochondrial matrix. Therefore, a better understanding of how manganese is addressed to the mitochondrial matrix, including using yeast as a model, could definitely help uncover the molecular basis behind the pathologies related to a decreased activity of SOD2.

### 3.4. Manganese-Related Neurodegenerative Disorders

Manganese is naturally present in our brain at approximately 0.25 µg/g wet weight [78]. However, under excessive and prolonged exposure, that occurs, for example, in welding and mining or in populations exposed to contaminated water, manganese can overaccumulate in the brain and lead to neurotoxicity. The most illustrative example of manganese neurotoxicity is the development of manganism, a Parkinson-like neurodegenerative disease that results from chronic exposure to manganese [79]. How manganese overaccumulation causes neurotoxicity is, however, not fully understood yet. This Section gathers two examples of neurodegenerative diseases for which manganese exposure is a risk factor, with molecular backgrounds at least partly elucidated from studies in yeast: early Parkinson or Kufor-Rakeb syndrome and prion-related pathologies.

#### 3.4.1. Ypk9p and Early Parkinson

The Kufor-Rakeb syndrome is a rare form of juvenile Parkinsonism whose featured symptoms are widespread neurodegeneration resulting in dementia and partial paralysis, among others. The gene responsible for this juvenile form of Parkinson has been identified as encoding the protein ATP13A2 [80]. Indeed, loss of function of ATP13A2 has been shown to lead to misfolding of the protein α-synuclein (α-syn), resulting in its incorporation in Lewy bodies [81], which are defined as abnormal protein aggregates that develop inside nerve cells and that contribute to the development of the pathology [82]. While this clearly indicates that ATP13A2 is a modifier of α-syn folding and subsequent toxicity, its exact function remains unknown.

ATP13A2 is predicted to encode a lysosomal P-type cation-transporting ATPase with undefined substrate specificity [83]. This human protein possesses a yeast ortholog: Ypk9p. In yeast, Ypk9p localizes to the vacuole [84,85]. While its exact function remains undefined, deletion of the corresponding gene leads to growth defects in the presence of manganese [85]. Interestingly, while re-expression of wild-type *YPK9* suppresses this Mn^2+^-induced toxicity, expression of the gene containing mutations corresponding to the disease-causing ones does not [84]. These findings suggest that Ypk9p and ATP13A2 play a key role in protecting cells from manganese toxicity through regulating the intracellular homeostasis of Mn^2+^ that likely contributes to the Kufor-Rakeb pathogenesis [38].

In the light of this newly-identified role of ATP13A2/Ypk9p, and in order to understand how they modulate α-syn toxicity, it is possible to heterologously produce α-syn, which possesses no clear yeast ortholog, in this host. Interestingly, when overexpressed in yeast, α-syn causes toxicity with hallmarks similar to the ones identified in mammalian neurons, making yeast a good model to study modulation of its toxicity through genetic and/or environmental factors [86]. In order to investigate this toxicity modulation mechanism, it was first verified that Ypk9p does not modulate the cellular accumulation of α-syn. Instead, it was shown that Ypk9p influences the cellular localization of α-syn [84]. Indeed, when overexpressed, α-syn localizes to intracellular inclusions, similarly to the Lewy bodies found in humans, and not to the plasma membrane anymore [86]. Besides, through analysis of the ability of Ypk9p containing the corresponding disease-causing mutations to suppress α-syn toxicity in the *ypk9Δ* strain, it appeared that both its vacuolar localization and ATPase activity are required to counter α-syn toxicity. The exact mechanism by which Ypk9p or ATP13A2 protect against α-syn toxicity remains to be further elucidated, including using this yeast model of the pathology. However, aggregation of α-syn has been studied in macaques and, interestingly, the amount of aggregated α-syn is higher in the gray matter of animals exposed to manganese. This observation strengthens the link between α-syn toxicity and manganese and suggests that α-syn may interact with manganese to trigger neuronal cell death [87].

**Figure 3:**
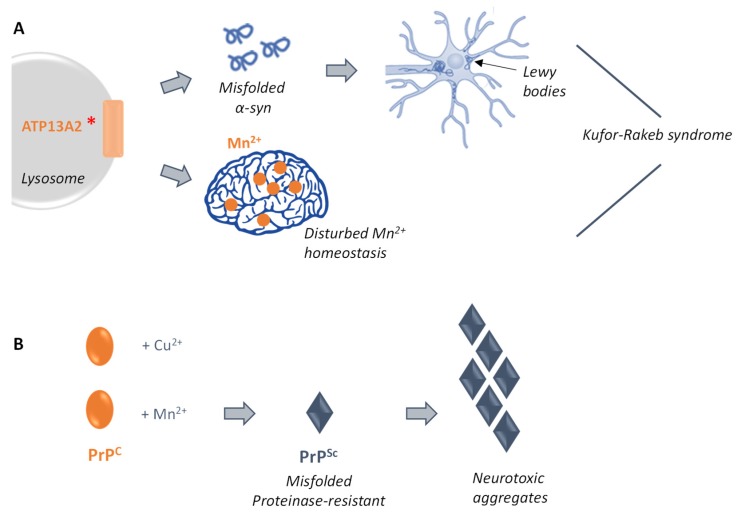
Mn^2+^-related molecular pathways associated with the development of two neurodegenerative diseases: the Kufor-Rakeb syndrome (**A**) and prion-related disorders (**B**). (**A**) Specific mutations within the human gene ATP13A2 have been shown to induce misfolding of the α-syn protein and its subsequent neurotoxic aggregation that leads to the formation of Lewy bodies in human neurons. In parallel, mutated ATP13A2 has been shown to directly disturb manganese homeostasis, thereby establishing a connection between genetics (α-syn and ATP13A2) and environmental (unbalanced manganese exposure) causes of neurodegeneration. (**B**) The human protein PrP interacts, in its functional isoform (PrP^C^), with copper, and also with manganese. This interaction with manganese has been suggested to induce the conversion of PrP^C^ to its proteinase-resistant counterpart PrP^Sc^. In this form, PrP forms neurotoxic aggregates, thereby leading to the prion-disease associated symptoms.

Manganese exposure is known for many years as an environmental risk factor linked to Parkinson’s disease [88]. Interestingly, the highlighted role of Ypk9p in protecting cells from manganese toxicity establishes a link between genetics (α-syn and Ypk9p) and environmental (unbalanced manganese exposure) causes of neurodegeneration (Figure 3A) [31,84,85]. Further studies on the function of Ypk9p/ATP13A2, mainly in terms of identification of the cellular mechanisms of toxicity and of new predisposition factors to early Parkinsonism, would definitely help define the molecular basis of Kufor–Rakeb syndrome and lead to the development of therapeutic alternatives.

#### 3.4.2. Prion-Related Diseases

The prion protein PrP, also known as CD230, is a key protein responsible for the development of a group of neurodegenerative diseases denominated as transmissible spongiform encephalopathies (TSEs) among mammals. Symptoms of such diseases range from altered memory and personality troubles to psychomotor defects. At the molecular level, a fundamental event that triggers the development of TSEs is the conversion of the normal isoform of the prion protein, PrP^C^, to its misfolded proteinase-resistant isoform, PrP^Sc^. Once misfolded, the protein forms aggregates in neurons, leading to neurodegeneration [89,90,91,92]. Naturally, PrP^C^ localizes to the cell membrane and has been shown to be involved in ligand uptake, cell signaling, cell adhesion, copper toxicity, and resistance to oxidative stress [92,93,94,95]. In this context, a better understanding of the mechanism behind this switch that converts the normal PrP isoform to its aggregating neurotoxic counterpart would definitely open new ways for possible treatment of the disease.

PrP^C^ is known to be a metalloprotein that binds copper in its normal function [96]. Apart from copper, PrP^C^ has been shown to interact with other transition metals, including manganese cations [97]. This interaction with manganese has been later directly demonstrated by Brazier et al. using isothermal titration calorimetry [98]. The putative involvement of the binding of manganese in the conversion mechanism and, hence, in the induced neurotoxicity has been further investigated in vivo in the yeast *Pichia pastoris* producing the murine PrP. In this context, it was shown that expression of PrP in yeast leads to a decrease of about 50% of its initial manganese content, indicating downregulation of the intracellular manganese level. Additionally, growing yeast cells in the presence of manganese in the extracellular medium induces the conversion of the PrP^C^ isoform to the disease-causing PrP^Sc^. Thereby, studies conducted in yeast have shown that PrP itself influences the manganese metabolism by a mechanism that still needs to be uncovered and that manganese binding to PrP leads to the predominance of the proteinase-resistant isoform, placing manganese as a risk factor to the development of TSEs (Figure 3B) [99,100].

Despite the evidence of manganese involvement in the switch mechanism, how these metal cations contribute to the formation of these aggregates remains unclear. To further study this mechanism, the influence of manganese and other divalent cations on the conversion and aggregation of one of the yeast prion proteins, Sup35p, was studied in *S. cerevisiae*. However, and surprisingly, Mn^2+^ does not bind to Sup35p and does not induce the formation of any aggregates [101].

The implication of manganese in the conversion mechanism of human PrP indicates that an excess of manganese in the environment might favor the formation of its proteinase-resistant isoform and, hence, the development of TSEs. Besides, while binding of manganese might of course not be the only way to generate toxic forms of PrP, it opens new putative perspectives of therapy, including chelation therapy specifically targeted at manganese to extend the lifespan of mammals suffering from prion disease. Such a strategy has been already tested in the mouse model of the disease and led to significant improvement in their survival [95].

## 4. Conclusions

Complete understanding of human pathologies at the molecular level is generally complex and puzzling. In order to gain insight into these disorders, one possibility is to take advantage of the fact that many pathology-related human genes possess a yeast functional ortholog. In combination with animal models used to validate observations made in yeast, the potential of yeast to study human disorders is vast. In parallel, new actors involved in yeast manganese homeostasis are still popping up in the literature, notably with the recent identification of Gdt1p as regulating manganese concentrations at the Golgi level. This review illustrates that the functional conservation from yeast to humans of proteins involved in manganese homeostasis makes yeast a relevant tool to uncover new aspects of poorly-understood human pathologies, and even to suggest new therapeutic approaches. Therefore, the increasing knowledge on yeast cation homeostasis and the constant identification of new rare disorders represent a large reservoir for further research on how cation homeostasis is controlled in eukaryotes and how to link it with the molecular mechanisms of such rare diseases.

## Figures and Tables

**Table 1:** Human transporters involved in manganese homeostasis that possess a yeast ortholog.

Human Transporter	Yeast Mn^2+^-Transporting Ortholog	Function in Human Cells
DMT1 [33]	Smf1p and Smf2p	Plasma membrane metal importer
		Involved in dietary manganese intake
NRAMP1 [20,34]	Smf1p and Smf2p	Plasma membrane metal importer
		Mostly expressed in the phagosome of macrophages where it limits the bioavailability of metals to invading microbes
SPCA1 and SPCA2 [35,36]	Pmr1p	Golgi-localized Ca^2+^ and Mn^2+^ P-type ATPases
		Involved in manganese detoxification and Golgi Mn^2+^ supply
TMEM165 [27,37]	Gdt1p	Golgi-localized Ca^2+^ and Mn^2+^ secondary transporter
		Putatively involved in manganese detoxification and Golgi Mn^2+^ supply
ATP13A2 [38]	Ypk9p	Putative lysosomal Mn^2+^ transporter
		Involved in manganese sequestration in the lysosome
ATP13A1 [29]	Cod1p	Mn^2+^ P-type ATPase at the endoplasmic reticulum
		Supplies the secretory pathway with manganese
